# Cancer treatment innovators discover Charles Darwin

**DOI:** 10.1093/emph/eoz018

**Published:** 2019-06-13

**Authors:** Robert Gatenby, Christopher Whelan

**Affiliations:** Cancer Biology and Evolution Program, Moffitt Cancer Center, Tampa, FL USA

With great fanfare, the Institute of Cancer Research announced formation of the ‘world’s first’ Darwinian Cancer Drug Programme within a new Centre for Cancer Discovery [1]. Supported by an initial investment of *£*75 million, the ICR plans to develop a ‘global center of expertise’ in cancer therapies that integrate evolutionary principles to improve outcomes. A particularly interesting area of investigation is their explicit goal to develop ‘anti-evolutionary’ drugs that seek to reduce the evolutionary speed of cancer cells as they adapt to treatment. This is a welcome and important addition to the growing number of cancer programs investigating the complex eco-evolutionary dynamics that govern drug resistance in clinical cancers.

For over a century, clinical cancer treatment research has focused on drug development. This line of investigation can trace its lineage to Paul Ehrlich, a Nobel Prize winner in Medicine who famously proposed the concept of ‘magic bullets’—an ideal therapeutic agent that could selectively kill cancer cells, leaving normal cells unharmed. The remarkable success of antibiotics in the 20th Century re-enforced the search for magic bullets in cancer therapy. In responding to this challenge, a world-wide drug development effort has produced an impressive array of new cancer treatment strategies and treatment agents. Unfortunately, magic bullets remain elusive despite a century of intense research since Ehrlich’s proposal.

In retrospect, the magic bullet hypothesis is deeply flawed and probably should be abandoned. The analogy of antibiotics for bacterial infection and chemotherapy for cancer, while intuitively appealing, is fundamentally incorrect. Bacteria have only minimal resemblance to eukaryotic mammalian host cells and have a limited genetic repertoire for adaptive strategies. Cancer cells, in contrast, have access to the vast information storehouse of the human genome to find strategies that overcome treatment. There is also the simple problem of arithmetic. A single gram of tumor may contain a billion cancer cells, and patients presenting with metastatic cancers typically have tumor burdens of tens or even hundreds of grams. Expectations that a single drug (or even group of drugs) could eradicate cancer populations larger than the number of humans currently on earth probably with similar and perhaps greater diversity seem unrealistic. Nevertheless, new and innovative treatments have led to greatly improved outcomes in local and regionally advanced cancer and modest extension of life expectancy in patients with metastatic cancers. Furthermore, unlike oncologic practice only one or two decades ago, at least one effective treatment agent now exists for nearly all forms of cancer.

Despite these advances, common disseminated malignancies, such as lung, prostate, breast, and pancreatic cancers, remain almost invariably fatal. Why? The simple answer is evolution. Cancer cells have a remarkable capacity to develop adaptive strategies to virtually every form of therapy. For example, there are currently 52 drugs [2] approved for treatment of metastatic prostate cancer. However, of the 35 000 men who will receive this diagnosis in the USA next year, not a single one is likely to be cured of his disease.

All of this has led some to reconsider the prevailing opinion that improved outcomes in metastatic cancers can be obtained only by developing new and better drugs. In particular, a few investigators have turned to our growing understanding of evolutionary dynamics that govern cancer treatment failure and critically examine the tactics with which the drugs are applied. Standard of care cancer therapy typically applies a drug or drugs at Maximum Tolerated Dose until progression. Analysis of this strategy in the context of the evolutionary dynamics of resistance has found it actually tends to accelerate the development of resistant cancer populations. This has led to the hypothesis that integration of evolutionary principles into treatment strategies can prevent or delay resistance, thus extending the duration of tumor control with currently available agents.

The Darwinian Cancer Drug Programme is the latest addition to this global research effort and will initially focus on three aspects of the intersection between evolutionary biology and cancer. The first area aims to develop a sequence of treatments in which the cellular adaptive strategy to the first treatment renders it more vulnerable to follow-on therapy. This very promising approach is built upon well-developed evolutionary theory. Described as an ‘evolutionary double bind’ [3] or ‘sucker’s gambit,’ [4] these evolutionary dynamics have been observed in a clinical trial in which cancer cells that successfully adapted to a p53 vaccine became substantially more responsive to chemotherapy [5]. More importantly, this approach focuses on a strategic, evolution-based linkage of the sequence therapies applied to each cancer patient. Currently drugs are evaluated and receive regulatory approval base on their demonstrated efficacy as a single agent. In other words, the activity of a drug critically depends on the ecological and evolutionary context of the tumor, in part generated by prior therapy, but this is not considered in either drug approval or protocol design when agents are used sequentially. Combinations of drugs are commonly used simultaneously but this is largely based on toxicity and pharmacodynamics considerations. Integrating evolutionary dynamics will require understanding of the mechanisms by which a cancer cell adapts to each agent and how these may enhance or diminish its ability to adapt to other agents when delivered in combination or sequence. The second primary area of research includes designing multidrug therapy to simultaneously target two different driver genes or to combine immunotherapy with other modalities. The third focus is identifying drugs that can slow the rate of cancer cell evolution. We assume this will focus on identifying drugs that decrease mutations and many cellular adaptive strategies simply require up or down regulation of existing normal genes. Furthermore, the speed of evolution is a generally increasing function of the phenotypic diversity and severity of selection forces in the environment. That is, even if a drug could reduce the mutation rate of a cancer cell, evolution will continue as a result of the cancer cell’s phenotypic plasticity (reaction norm) and the steep slope on the fitness landscape produced by application of any cytotoxic agent.

These lines of investigation are very welcome and it will be fascinating to see how the insights obtained through that activities of this new institute alter conventional cancer therapy over the coming years. That said, a bit of caution is in order. In their announcement, the institute claims that its scientists will ‘outsmart’ [1] cancer to improve cure rates. It is wise to remember the sage words attributed [6] to Leslie Orgel: ‘evolution is cleverer than you are’. To which we would add, based on personal experiences, ‘evolution has an ironic sense of humor and is particularly fond of embarrassing the unwary and the overconfident’.

Finally, we hope the investigation of cancer evolution can free itself of its genetic shackles. Darwinian dynamics in cancer, as in all of nature, are fundamentally governed by environmental selection forces interacting with the cellular phenotypes. Genes, as the ‘mechanism of inheritance’ for adaptive strategies, serve as a history of prior evolution but are not necessarily the fundamental drivers of those dynamics—in other words, they are more often the consequences rather than the cause of a cancer cell’s resistance to treatment. It is important to recognize that an evolving cancer cell benefits most from access to the vast information storehouse of the human genome which includes, among other things, extensive repertoires for xenobiotic metabolism and alternative expression patterns that can readily overcome many, perhaps most, current therapies without mutations.

That said, the formation of the Darwinian Cancer Drug Programme is part of an encouraging trend in cancer treatment investigations that, in addition to drug development, identifies new tactics based on evolutionary first principles to use existing drugs more effectively than the standard continuous administration at maximum tolerated dose until progression.

## FUNDING

This work is supported by NIH/National Cancer Institute (NCI) R01CA170595, Application of Evolutionary Principles to Maintain Cancer Control (PQ21), and NIH/NCI U54CA143970-05 [Physical Science Oncology Network (PSON)] “Cancer as a complex adaptive system.”

**Conflict of interest**: None declared.
